# Experimental Study on Shear Strengthening of Reinforced Concrete Beams by Fabric-Reinforced Cementitious Matrix

**DOI:** 10.3390/ma17174336

**Published:** 2024-09-01

**Authors:** Chanseo Jung, Yujae Seo, Junseo Hong, Jinhyeong Heo, Hae-Chang Cho, Hyunjin Ju

**Affiliations:** 1 Department of Architecture and Architectural Engineering, Hankyong National University, Jungang-ro 327, Anseong 17579, Republic of Korea; wjdckstj@hknu.ac.kr (C.J.); wnstj8554@hknu.ac.kr (J.H.); jun656554@hknu.ac.kr (J.H.); 2 Architecture Convergence Laboratory of Industry-Academic Cooperation Foundation, Hankyong National University, Jungang-ro 327, Anseong 17579, Republic of Korea; tjdbwo@hknu.ac.kr; 3 Technology Center, Dream Structural Engineers Co., Ltd., Hwaseong 18471, Republic of Korea; hc.cho@dreamse.co.kr; 4 School of Architecture and Design Convergence, Hankyong National University, Jungang-ro 327, Anseong 17579, Republic of Korea

**Keywords:** shear strength, fabric-reinforced cementitious matrix, DIC analysis, shear behavior, bond capacity

## Abstract

In this study, an experiment was conducted to investigate the shear performance of reinforced concrete (RC) beams strengthened using fabric-reinforced cementitious matrices (FRCM). Four reinforced concrete beams, including a control specimen, were fabricated, and the shear strengthening effect of the FRCM was investigated on eight shear specimens, with the strengthening type and shear reinforcement as key variables. In particular, the digital image correlation (DIC) technique was applied to closely analyze the deformation of reinforced concrete beams subjected to shear forces. The average shear strain–shear stress curve of each specimen was derived, and the contributions of shear and bending to the vertical deflection and the change in the principal strain angle with increasing shear force were analyzed. The experiment results showed that all specimens failed with diagonal cracks within the shear span. In the specimens without shear reinforcement, the shear strength increased by up to 65% according to the FRCM strengthening, while in the specimens with shear reinforcement, only the sided bond strengthened specimen showed a strength increase of 16% compared to the control specimen. Based on displacement data of the DIC, it was confirmed that FRCM strengthening can control the deformation of the RC beam. To evaluate the shear strength of the FRCM-strengthened RC beams, a shear strength model was proposed by considering the contributions of the concrete section, shear reinforcement, and FRCM. The proposed model was capable of reasonably evaluating the shear strength of RC beams strengthened with FRCM, considering the shear contribution of FRCM and bond capacity between FRCM and concrete substrate, in which the shear strength of specimens was underestimated by 28% to 35%.

## 1. Introduction

Building structures deteriorate over time owing to environmental factors, and their strength is continuously reduced owing to a lack of maintenance and management [1]. To ensure the safe use of structures, it is necessary to rehabilitate their degraded strength. Various studies have been conducted on structural strengthening methods. Among the traditional strengthening methods, the FRP (Fiber-Reinforced Polymer) method is used to attach fibers to substrates with polymer-based adhesives, which have a high strength-to-weight ratio, high corrosion resistance, and excellent constructability [2,3]. However, FRP materials have the disadvantages of low fire resistance and difficulty of application in wet environments [4]. FRCM (Fiber-Reinforced Cementitious Mortar) or TRM (Textile-Reinforced Mortar) is a material that addresses the drawbacks of FRP by using an inorganic cementitious binder instead of a polymer-based adhesive [5,6]. FRCM exhibits excellent compatibility with structural materials such as cement bricks and concrete, and its breathability effectively allows moisture to escape, preventing moisture-related damage [7,8]. Additionally, unlike polymer-based adhesives, FRCM has excellent fire resistance and can be applied to surfaces with moisture [9].

Previous studies to investigate the strengthening effect of FRCM focused on bond and flexural behaviors of FRCM applied to reinforced concrete beams [10,11], and it is required to study the shear strengthening effect of FRCM on existing reinforced concrete beams. The shear behavior of reinforced concrete members is relatively complicated, and the failure mode in shear is quite brittle; thus, many researchers have tried to investigate the shear behavior and suggested analytical models [12,13,14]. An experimental study was conducted to investigate the shear strengthening effects of FRCM. In an experimental study to verify the shear reinforcement performance of FRCM, Loreto et al. [15] conducted an experiment using the number of fiber stacks of U-shaped FRCM as a key variable. The experimental results confirmed a shear strength enhancement effect of at least 121% and up to 161% and reported that the increase in strength is not proportional to the number of fabric layers. Tetta et al. [16] conducted experiments on a control RC specimen and eight TRM- and five FRP-strengthened specimens with three types of fiber attachments (double-sided attachment, U-shape, and full wrapping) and the number of fiber layers as variables. The results showed that the strengthening effect of the FRP was better than that of the TRM when a single layer of fiber was used. However, for multiple layers of fabric, the strengthening effect of TRM was greater than that of FRP, and the full wrapping and U-shaped types showed a better strengthening effect than the side attachment. The failure mode depends on the number of fiber layers. When a single layer of fabric was used for strengthening, sliding at the surface of the fabric occurred at failure, while multiple layers of fabric resulted in interfacial delamination between the concrete and FRCM. Awani et al. [17] conducted experimental and analytical studies with the presence and amount of shear reinforcement as key variables. All the reinforced specimens failed by delamination of the FRCM from the concrete substrate, and the deformation of the shear reinforcement was reduced by FRCM strengthening. It was also found that the strengthening effect was similar, regardless of the amount of shear reinforcement. Trapko et al. [18] conducted experiments with fiber orientation as a variable, and the results showed that the specimen reinforced at 90 degrees exhibited the greatest reinforcement effect. Tzoura and Triantafillou [19] conducted cyclic load tests on T-beams reinforced with concrete FRP and FRCM jackets, using mechanical anchors and fiber unit weight as variables. The results showed that the anchor system enhanced the effectiveness of both FRCM and FRP and that the reinforcement effect increased with the fiber unit weight. Blanksvärd et al. [20] conducted experiments with the spacing of fiber bundles as a variable. The results showed that as the spacing of FRCM fibers was reduced, the load at which cracks occurred increased. Azam and Soudki [21] used both glass and carbon fibers in their experiments, varying the spacing of the carbon fibers to test three different fiber configurations. The results showed that the reinforcement effect of the carbon fibers was the most significant. Escrig et al. [22] conducted experiments with different types of fibers (PBO, basalt, glass, and carbon) as variables. The results showed that PBO fibers had the most significant effect, while basalt and glass fibers produced similar results. In contrast, carbon fibers yielded inconsistent results. These findings, which are independent of the tensile strength or elastic modulus of the fibers observed in the previous two studies, suggest that factors other than fiber type influence the outcomes [23].

An experimental study was conducted to investigate the shear performance of reinforced concrete (RC) beams strengthened by carbon FRCM in the presence or absence of shear reinforcement, with the strengthening type of the FRCM as the key variable. Although it is relatively easy to derive the relationship between the increase in shear strength and deformation, such as vertical deflection in the experiment, the data measured by conventional measuring instruments, including strain gauges and linear variable displacement transducers (LVDTs), are limited to closely analyzing the deformation caused by crack propagation and the shear behavior of reinforced concrete beams [24,25]. Therefore, the digital image correlation (DIC) technique was applied to characterize the shear behavior of FRCM-strengthened RC beams. In the shear strengthening of FRCM, the bond length between the FRCM and concrete should be sufficient to secure the strengthening effect [26,27]. However, it is not easy to make the bond length sufficient compared to flexural strengthening, in which the FRCM can be attached to the substrate in the longitudinal direction because of the dimensions of the beams. The shear bond length is relatively short, and if a mechanical anchorage device is not applied, the bond capacity between the FRCM and the concrete substrate governs the shear behavior and strength of the FRCM-strengthened RC beams. 

Strength is reached before the fiber-breaking strength is reached, and the reinforcement is likely to detach from the base material [28,29]. Therefore, the strength of the reinforcement is dominated by the attachment strength rather than the breaking of the fiber. This study aimed to evaluate the strength of RC members reinforced with FRCM by introducing an attachment reduction factor.

## 2. Experimental Scheme

### 2.1. Specimen Detail

**Figure 1:**
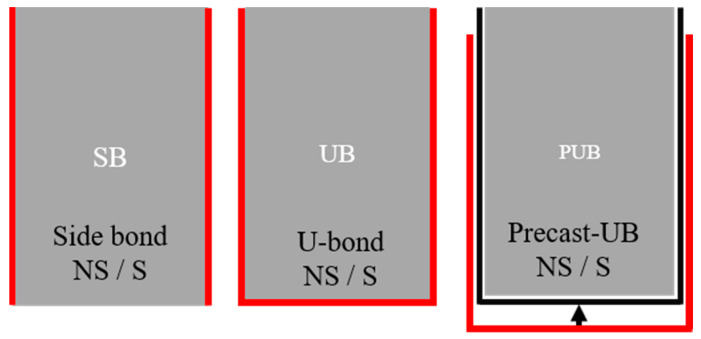
FRCM strengthening type.

The main experimental variables in this study were the presence or absence of shear reinforcement and the type of FRCM strengthening. Four RC beams, including the control RC beam, were strengthened by FRCM at both ends; thus, eight specimens were tested. Figure 1 shows the FRCM strengthening methods used in this study. The FRCM strengthening included a single layer of carbon fabric. The specimen naming refers to control (C), side-bond (SB), U-bonding (UB), and precast-U-bonding (PUB) according to the strengthening type of FRCM, and the letters after the test name, NS and S represent non-stirrup and stirrup, respectively. In other words, the SB specimen was a type of FRCM strengthening on the side of the beam, whereas the UB specimen was a type of FRCM strengthening on the side and bottom of the beam in a U-shape. PUB is a specimen strengthened by FRCM that was invented in this study for better construction, in which FRCM strengthening was applied with U-shaped reinforcement; however, the external FRCM panel was prefabricated and attached to the RC beam by applying a filling mortar between the prefabricated FRCM panel and the concrete substrate [30]. 

**Figure 2:**
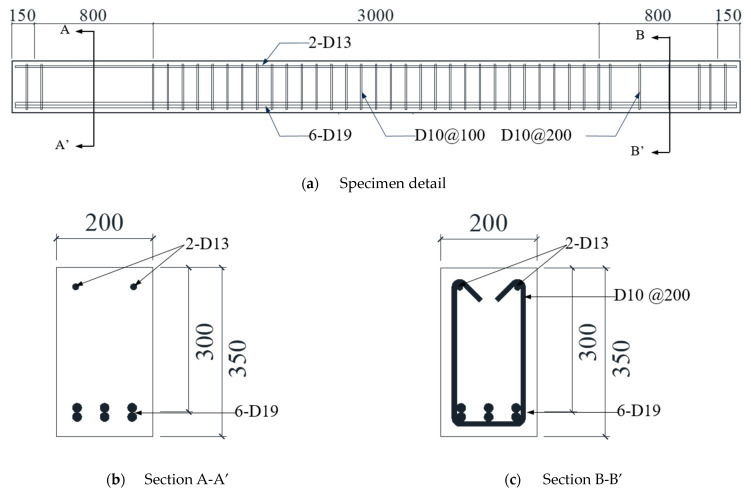
Specimen detail (unit: mm).

Figure 2 shows the specimen details, including the cross-section and reinforcement. The total span length of the beams was 4900 mm, with a cross-sectional width of 200 mm, height of 350 mm, and effective depth (d) of 300 mm. Two deformed bars with a diameter of 12.7 mm designated as D13 were placed at the top of the beam and six deformed bars with a diameter of 19.1 mm designated as D19 were placed at the bottom, with a longitudinal tensile reinforcement ratio of 2.87%. The sectional areas of D13 and D19 are 126.7 mm^2^ and 198.6 mm^2^, respectively. A shear span length of 800 mm at both ends of the beam was set as the test region, with a shear span ratio (*a*/*d*) of 2.66. The shear stirrups with a diameter of 9.53 mm, designated as D10, were placed at a spacing of 200 mm in the test region. The section area of D10 is 71.3 mm^2^, and the shear reinforcement ratio is 0.35%. The sectional area of D10 was 71.3 mm^2^. In the mid-span of the test region, a length of 3000 mm, D10 stirrups were placed at 100 mm intervals, as shown in Figure 2a, to prevent shear failure in the region. The strengths of the concrete and rebars used in the RC beams are summarized in Table 1. The compressive strength ($f_{ck}$) was 36.84 MPa, and the splitting tensile strength ($f_{sp}$) was 2.18 MPa, as measured on the same day as the beam tests. Tensile tests of the rebars showed that the yield strengths ($f_{y}$) of D10, D13, and D19 were 483, 526, and 557 MPa, respectively. The ultimate strengths ($f_{u}$) of D10, D13, and D19 were 621, 635, and 701 MPa, respectively.

### 2.2. FRCM Strengthening

**Figure 3:**
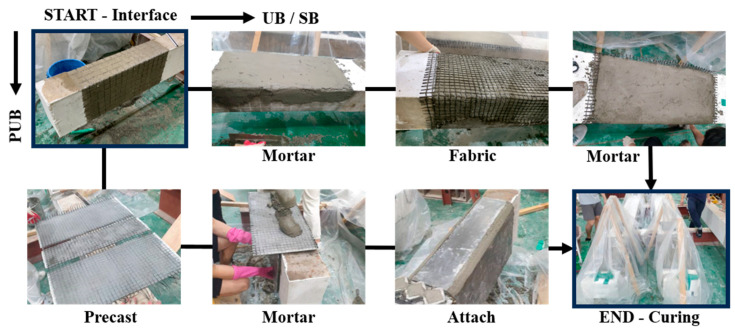
FRCM strengthening process.

Figure 3 illustrates the FRCM strengthening process. First, the surface of the concrete beam was grooved using a grinder to improve the adhesion of FRCM to the mortar matrix and concrete. Then, the first layer of mortar is applied, the fabric is embedded in the first layer of mortar, and the last layer of mortar is applied and cured to complete the strengthening. For the PUB specimens, prefabricated FRCM panels, which were made by mixing mortar and fibers as a plate in advance, were attached to the concrete substrate by filling mortar between the panel and concrete. 

Table 2 summarizes the material properties of the fabric and mortar used in the FRCM. The cube compressive and flexural tensile specimens were fabricated with the same mortar used for each FRCM strengthening. The material test showed that the average compressive strength ($f_{cm}$) and flexural tensile strength of the mortar used for side-bond (SB) were 59 MPa and 7.41 MPa, respectively, for U-bond (UB) were 62.7 MPa and 6.51 MPa, and for Precast-UB (PUB) were 41.58 MPa and 5.86 MPa, respectively. The mortar mix consists of cement (550 kg/m^3^), silica fume (28 kg/m^3^), wasted glass powder (110 kg/m^3^), recycled fine aggregate (1487 kg/m^3^), water (206 kg/m^3^), superplasticizer (14 kg/m^3^), and deformer (4 kg/m^3^). For construction sustainability, wasted glass power and recycled fine aggregate were incorporated into the mortar mix, and the recycled materials could be sensitive to lab circumstances such as temperature and moisture. The specimens SB, UB, and PUB were fabricated on different days; thus, the material properties have some variation according to the strengthening type. The carbon fabric used in this study had a spacing of 20 mm in the orthogonal direction, and the cross-sectional area of the fiber ($A_{f}$) was 0.838 mm^2^. A direct tensile test of the fiber showed that the elastic modulus ($E_{f}$) and tensile strength ($f_{fu}$) were 184 MPa and 1962 MPa, respectively. 

### 2.3. Loading and Measurement

**Figure 4:**
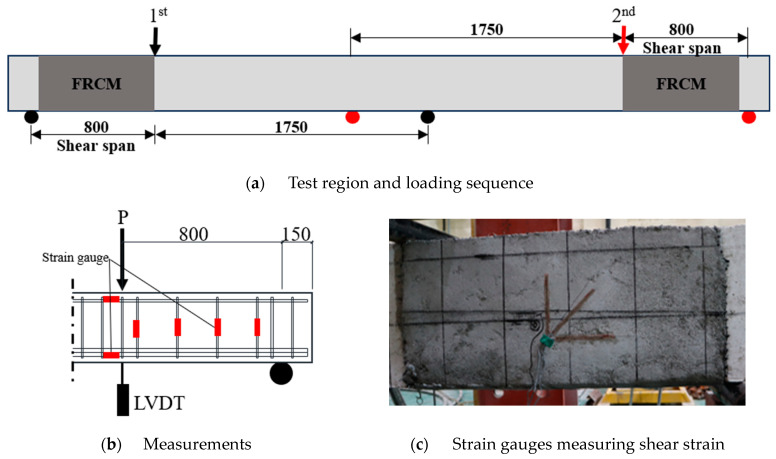
Experiment procedure (unit: mm).

The experiment was conducted using a 1000 kN actuator at a rate of 1 mm/min. As shown in Figure 4a, the end of the RC beam without shear reinforcement was tested first, followed by the other end region with shear reinforcement [31]. These are the NS- and S-series specimens, respectively. The total span between the supports was 2550 mm, and the shear span of the shear failure region was 800 mm. Linear variable displacement transducers (LVDTs) were installed under the loading points to measure the vertical deflection of the beams, and strain gauges were installed on all the shear reinforcements in the test region, as shown in Figure 4b. To measure the shear strain in the concrete web section of the test specimen, rosette-type concrete gauges were attached in the horizontal, transverse, and 45° diagonal directions to the surface of the specimen, considering the anticipated path of the critical shear crack, as shown in Figure 4c.

**Figure 5:**
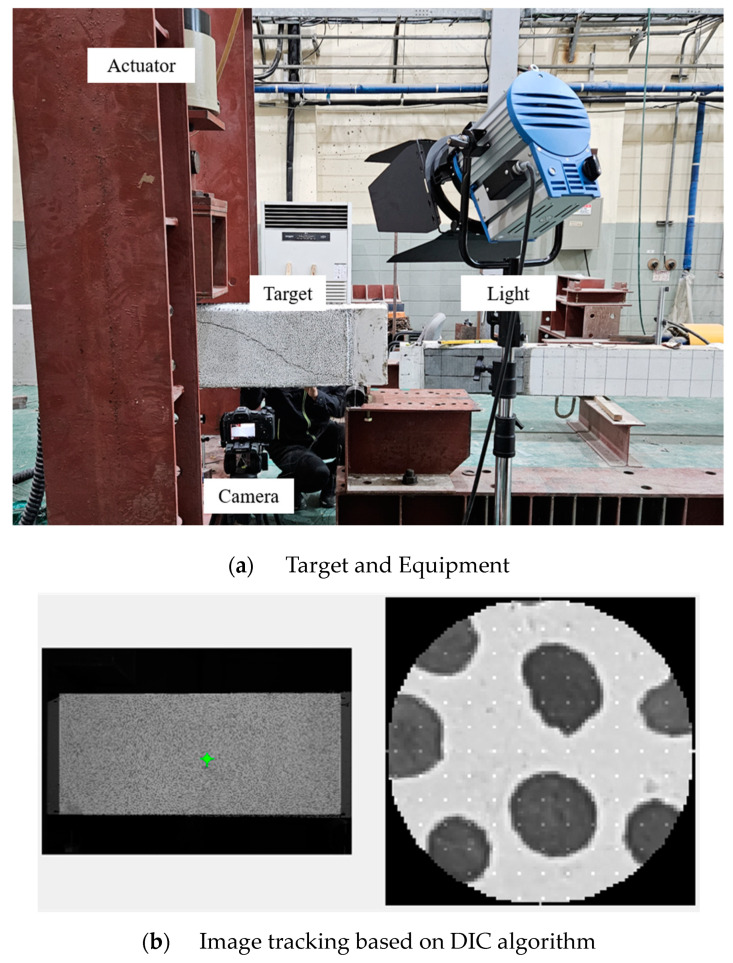
Digital image correlation process.

The measurement setup for the digital image correlation (DIC) technique consisted of a camera, light, and a target region of irregular speckles, as shown in Figure 5. Because the DIC analysis algorithm tracks the displacement within the shear span based on a specific initial shape within the target, irregular speckles are required on the face of the measurement area to achieve the desired accuracy. In addition, a high-pixel camera is required to ensure that each dot is distinguishable in the enlarged image, and lighting is necessary for a clear contrast between the speckles and the background. An initial image was captured before the test began, and image data were acquired every 60 s after the loading started, that is, every 1 mm of displacement. It is important to stabilize the camera because the displacement data cannot be accurately analyzed if the camera frame moves during the test. The position of each speckle was tracked by a MATLAB-based image analysis algorithm, and the displacements and strains were calculated in the region of interest [32,33]. In this study, the DIC technique was applied to analyze the shear behavior of reinforced concrete beams, including the local shear strain along diagonal tensile cracks, average shear strain within the shear span, and variation in the principal strain direction according to the shear force.

## 3. Experimental Results

### 3.1. Failure Mode and Crack Pattern

**Figure 6:**
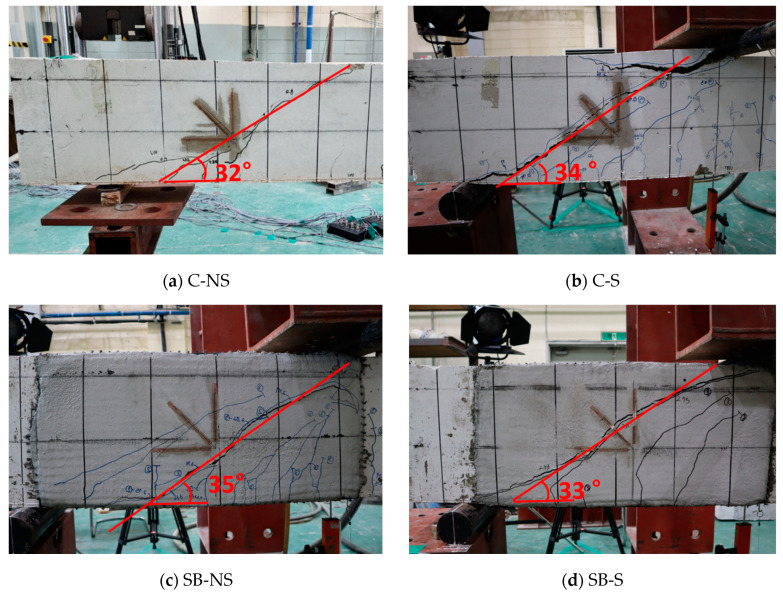

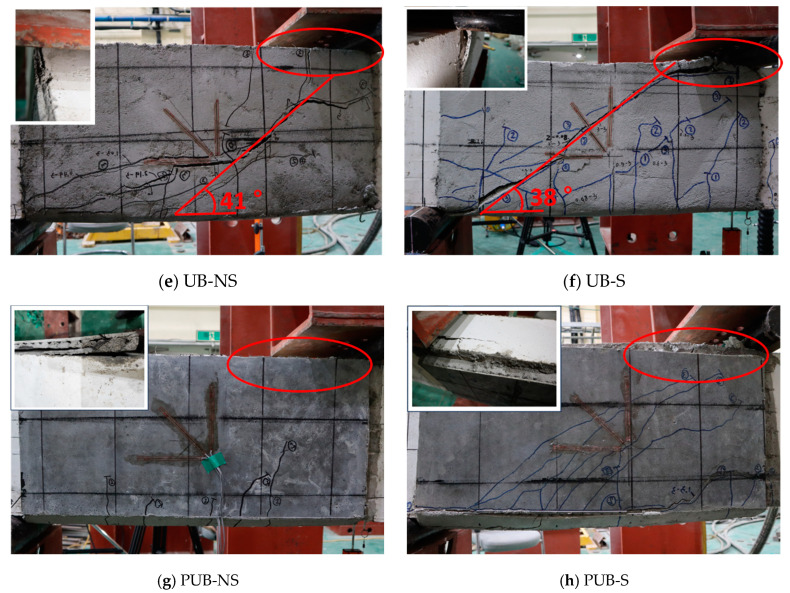
Crack patterns at failure.

Figure 6 shows the crack patterns of the specimens at the end of the test. All the specimens exhibited a shear-dominant failure mode, with the control specimen C-NS exhibiting only diagonal cracks without any flexural cracks in the shear span. In the FRCM-strengthened SB-S specimen with shear reinforcement, several flexural cracks developed, whereas the NS series without shear reinforcement failed in a brittle manner shortly after the cracks were identified. As shown in Figure 6e,f, the UB-NS and UB-S specimens with U-shaped FRCM exhibited a loss of bonds at the concrete-to-mortar and mortar-to-mortar interfaces, respectively, on the top of the beams at the ultimate state. In addition, the PUB specimens with the prefabricated FRCM panels showed that the FRCM panel delaminated from the surface of the concrete substrate during the test, as shown in Figure 6g,h. Critical shear cracks occurred at angles of 32° and 34° in the longitudinal direction in the C-NS and C-S specimens, respectively, while crack angles of 34° and 35° occurred in the SB-NS and SB-S specimens, respectively. Additionally, the shear crack angles were similar for the C- and SB-series specimens, whereas the UB-NS and UB-S specimens exhibited significantly larger shear crack angles of 41° and 38°, respectively. 

### 3.2. Load–Deflection Relationship

**Figure 7:**
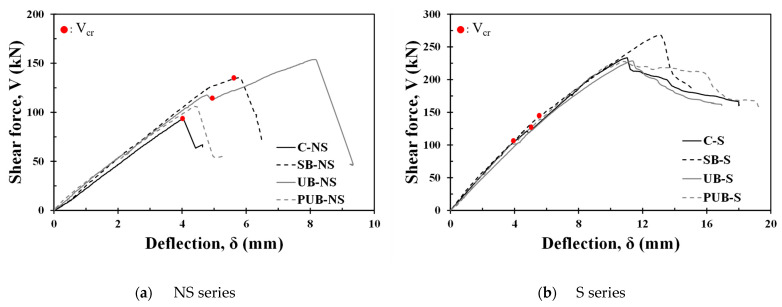
Shear force versus deflection curves.

Figure 7 shows the shear force–deflection relationships of all specimens tested in this study, and the experimental results are summarized in Table 3. The maximum strength of the C-NS specimen without shear reinforcement was 93.3 kN, and shear strength improvement was confirmed in all the FRCM strengthening types of the NS series specimens. The shear strengths of the SB-NS, UB-NS, and PUB-NS specimens were 135 kN, 153.8 kN, and 106 kN, respectively, and the strength improvement effects were 45%, 65%, and 14%, respectively, compared with those of the C-NS specimen. Therefore, the UB-NS specimen with U-shaped FRCM was the most effective for strengthening the shear capacity of RC beams without shear reinforcement, and the prefabricated FRCM panel type was not fully effective because the bond capacity might not have been fully activated at the interface between the FRCM panel and the concrete substrate. The C-NS and SB-NS specimens without shear reinforcement failed at the shear cracking strength of 93.3 and 134.4 kN, respectively. The shear crack strength of the UB-NS specimen was 112 kN, which was lower than that of the SB-NS specimen; however, it exhibited additional shear resistance after cracking, followed by brittle failure. This suggests that U-shaped FRCM strengthening is more effective than side-bond strengthening for crack control and load-carrying capacity. 

Among the S-series specimens with shear reinforcement, the control specimen C-S showed an ultimate shear strength of 233.4 kN, and the shear strength of the side-bond FRCM specimen SB-S was 268 kN, indicating a strength enhancement effect of approximately 15%. However, the UB-S and PUB-S specimens exhibited almost the same shear strength as the C-S specimen, indicating that the shear strengthening effect was not confirmed by the prefabricated FRCM panel. Diagonal shear cracks occurred in the C-S, SB-S, and UB-S specimens with shear reinforcement at 105, 141, and 122.4 kN, respectively. Although the trend of cracking strengths was similar to that of specimens without shear reinforcement, the maximum strength of the UB-S specimen was comparable to that of the C-S specimen. For the PUB-S specimen, no failure-inducing shear cracks were observed on the FRCM reinforcement surface, as shown in Figure 6g,h, and cracks occurred in the mortar between the FRCM panel and concrete member. This indicates that the RC member and FRCM panel were not fully bonded; therefore, an increase in the shear strength could not be achieved. For the UB-S specimen, detachment of the FRCM was observed at failure, as shown in Figure 6f. It appears that the bond between the FRCM and the concrete beam was lost before the shear strength was reached because the bond strength was insufficient at the top of the FRCM during loading.

### 3.3. Shear Reinforcement Strain

**Figure 8:**
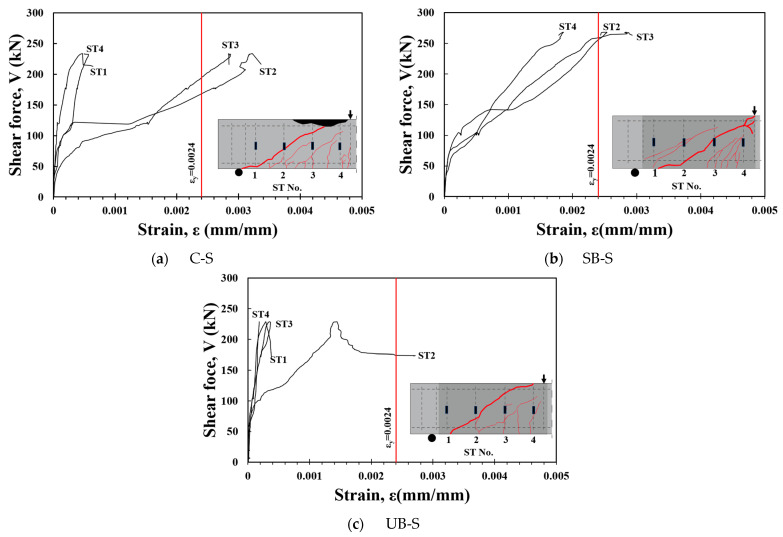
Strain of shear reinforcement.

Strain gauges were attached to all stirrups in the test region of the specimens where the shear reinforcements were placed to monitor the strain of the shear reinforcement. As shown in Figure 8a, the strain of the shear reinforcement in the C-S specimen shows that the shear reinforcement reached the yield strain before the shear strength was achieved. Figure 8b shows that the shear reinforcement strain in the SB-S specimen reached the yield strain as the shear force increased. Subsequently, the shear force increased further before reaching the maximum strength. This means that the maximum shear strength of the SB-S specimen was achieved when the strength of the FRCM was well developed after the shear reinforcement yielded. However, in the UB-S specimen with U-shaped strengthening, the strain of the shear reinforcement was measured to be less than the yield strain at the peak strength, as shown in Figure 8c. This might be due to the insufficient bond strength between the FRCM and the concrete member, which caused the FRCM to detach prematurely with an increase in load. It was presumed that the shear force resisted by the FRCM was instantaneously transferred to the shear reinforcement, resulting in a rapid increase in the strain of the shear reinforcement after the maximum strength was reached. 

### 3.4. Digital Image Correlation Analysis

**Figure 9:**
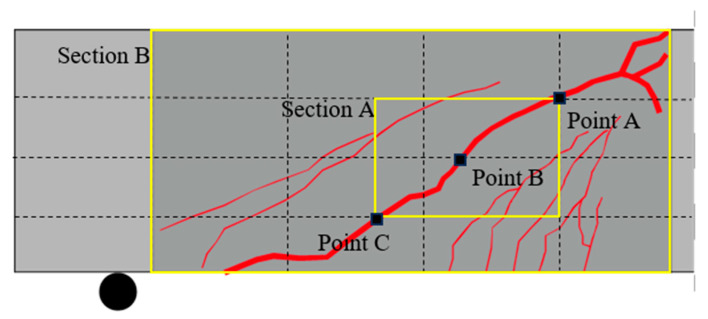
Region of interest for DIC analysis.

Figure 9 shows the region of interest where DIC analysis was performed. The height of the beam was divided into four equal parts, and the points where the vertical line and shear crack overlapped were set as Points A, B, and C. Section A was defined as the rectangular area with Points A and C as the corners. The entire shear span was defined as in Section B. For the DIC analysis, the surface must deform from point to point. The PUB specimen was excluded from the DIC analysis because it did not produce displacement due to its detachment from the RC beam. 

In the DIC analysis, the strain data in the horizontal and vertical directions were obtained by tracking the change in the position of the speckles by comparing the initial and updated images. The strains in the vertical, horizontal, and shear directions ($\varepsilon_{xx}$, $\varepsilon_{yy}$, and $\gamma_{xy}$) were calculated as follows:

(1) $$\varepsilon_{xx}=\frac{1}{2}\left(2\frac{du}{dx}+{\left(\frac{du}{dx}\right)}^{2}+{\left(\frac{dv}{dx}\right)}^{2}\right)$$

(2) $$\varepsilon_{yy}=\frac{1}{2}\left(2\frac{dv}{dy}+{\left(\frac{du}{dy}\right)}^{2}+{\left(\frac{dv}{dy}\right)}^{2}\right)$$

(3) $$\gamma_{xy}=\frac{1}{2}\left(2\frac{du}{dy}+\frac{dv}{dx}+\frac{du}{dx}\frac{du}{dy}+\frac{dv}{dx}\frac{dv}{dy}\right)$$

where u and v are the horizontal and vertical displacements, respectively, and the strain data were calculated by considering the amount of displacement change at each point. 

**Figure 10:**
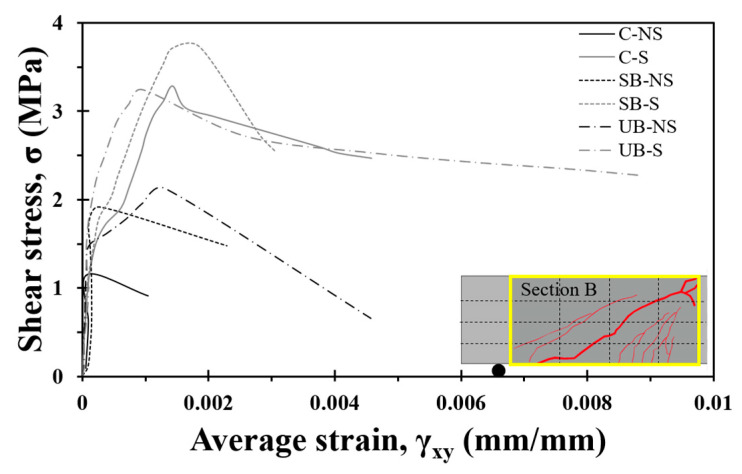
Average shear strain versus shear stress curves.

Figure 10 shows the average shear strain of the specimens calculated using Equation (3) for Section B versus the shear stress, defined as the shear force divided by $b_{w}d$. The average shear strain can vary according to the global or localized regions considered. For the C-NS and SB-NS specimens, there was no significant change in the stiffness until the maximum load was reached, and the shear strain increased rapidly after the cracking shear strength, leading to failure. For the UB-NS specimen, the stiffness decreased significantly after crack initiation; however, the load increased linearly until the ultimate strength was reached, after which the shear strain increased rapidly. The S-series specimens with shear reinforcement exhibited a linear trend of increasing strain from cracking to the ultimate strength. The S series did not show much stiffness degradation after shear cracking compared with the NS series and showed a relatively large deformation capacity. In addition, the FRCM-strengthened S specimens showed greater stiffness after shear cracking than the C-S specimens. In particular, the deformation control effect and stiffness of the UB-S were greater than those of the SB-S. Overall, based on the comparison of the average shear strains of the specimens, UB strengthening appeared to be the most effective in controlling shear deformation.

**Figure 11:**
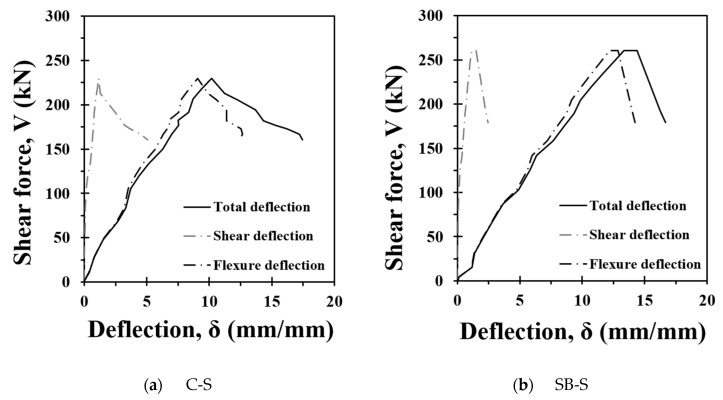
Shear and flexure deflection curves.

Figure 11 shows the contributions of shear and flexure to the vertical deflection. The contribution of shear to the deflection was calculated by multiplying the average shear strain ($\gamma_{xy}$) derived in Section B by the shear span length (a). For the total deflection, the vertical displacement data of the bottom of the beam at the loading point were used, and the deflection due to flexure was calculated by subtracting the deflection due to shear from the total deflection. For both the C-S and SB-S specimens, the deflection was mainly caused by the fundamental deformation until the shear crack occurred, and the shear deflection increased linearly after the shear crack occurred. In the C-S specimens, the shear deflection increased more rapidly after reaching the maximum strength. The SB-S specimen showed a similar trend; however, because the shear deformation was controlled by the FRCM, the increase in deflection owing to shear after reaching the ultimate strength was relatively small.

When the specimens resist a shear force, rosette-type strain gauge data can be utilized to analyze the principal strain angle within the shear span. However, if the critical crack does not pass through the gauges, the measured strain can be underestimated. Thus, in this study, the principal strain angle (θ) was calculated based on the strain data extracted from the DIC analysis as follows:

(4) $$\theta =\frac{1}{2}\tan^{-1}\left(\frac{\gamma_{xy}}{\varepsilon_{xx}-\varepsilon_{yy}}\right)$$

**Figure 12:**
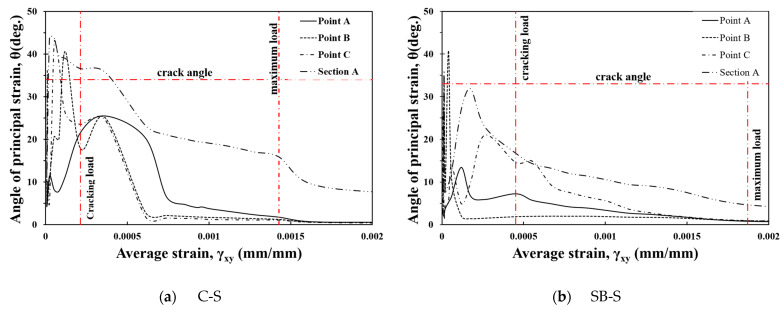
Principal strain angle.

The principal strain angle (θ) can be derived based on the calculated strain range, as in the previous calculation of the shear strain. Figure 12a,b show the relationship between the shear strain ($\gamma_{xy}$) and the principal strain angle (θ) for the C-S and SB-S specimens, respectively. Here, Section A and Points A, B, and C, indicated in Figure 9, were set as the measurement ranges to analyze the average principal strain angles that include the shear cracks leading to failure and the principal strain angles that occur locally at the crack surface. 

Although there are differences in the measurement range, in general, the principal stress angle is estimated to be approximately 35–45°at a small shear strain, and the principal strain angle (θ) decreases rapidly after a crack occurs. The crack appeared relatively later, and the strain angle remained low in the FRCM-strengthened SB-S specimen compared to the C-S specimen. This is because cracks cannot be observed on the concrete surface owing to the FRCM, and they are observed on the FRCM surface after the shear force is transmitted to both the RC member and the FRCM. The red horizontal dotted lines in Figure 12 represent the crack angles observed in the crack pattern of the test specimen. Compared to the observed crack angles, the principal strain angles are generally somewhat different but tend to be similar to the principal strain angle (θ) at crack initiation and are most similar to the average principal strain angle calculated in Section A. The local principal strain angles calculated at Points A, B, and C differed significantly from the average principal stress angles. Thus, it can be presumed that the principal strain angle does not coincide with the critical shear crack angle, and the differences between the average and local principal strain angles are closely related to the shear resistance stress at the crack surface, which is explained by the aggregate engagement [34]. 

## 4. Evaluation of Shear Capacity

### 4.1. Evaluation Model

The shear strength of the reinforced concrete beam specimens can be improved by FRCM strengthening. However, sufficient bond capacity between FRCM and concrete beams must be ensured for effective strengthening. If the bond between the concrete beam and FRCM is assumed to be perfect, the failure of the beam is determined by the ultimate tensile strength of the fabric embedded in the FRCM. In this study, a strength evaluation model is proposed that assumes a partial bond capacity based on the tensile strength of the fabric. 

When a reinforced concrete beam with shear reinforcement is strengthened by an FRCM, the shear strength of the beam ($V_{n}$) is calculated as the sum of the shear strength contributions of the concrete ($V_{c}$), shear reinforcement ($V_{s}$), and FRCM ($V_{f}$), as in ACI 549 [35]:

(5) $$V_{n}=V_{c}+V_{s}+V_{f}$$

The shear strength contributions of the concrete and shear reinforcement were calculated according to ACI 318 [36] as follows:

(6) $$V_{c}=\frac{1}{6}\sqrt{f_{ck}}b_{w}d$$

(7) $$V_{s}=V_{sy}=\frac{A_{v}f_{y}d}{s}$$

where $V_{sy}$ is the shear strength of the shear reinforcement, assuming the yielding of the reinforcement. In addition, $f_{ck}$ is the compressive strength of the concrete, $b_{w}$ and d are the width and effective depth of the beam, respectively, and $A_{v}$, $f_{y}$, and s are the cross-sectional area, yield strength, and spacing of the shear reinforcement, respectively. 

When an FRCM-strengthened RC beam with shear reinforcement resists shear forces, it can be assumed that the shear force deduced from the shear strength of concrete ($V_{c}$) should be resisted by the shear reinforcement and FRCM, considering the stiffness ratio of the shear reinforcement and FRCM. If the shear reinforcement yields, $V_{sy}$ can be used because of the ductile behavior of the reinforcement, and the additional shear force can be considered to be resisted by the maximum strength of the FRCM. However, if the maximum strength of the FRCM is reached before the yielding of the shear reinforcement, the maximum shear strength ($V_{n}$) will also be reached before the yielding of the shear reinforcement, and the resistance of the FRCM will be lost, causing the shear reinforcement to yield. In this case, assuming that the FRCM and shear reinforcement are subjected to the same strain, the shear stiffnesses $K_{f}$ and $K_{s}$ are defined as follows:

(8) $$K_{f}=\frac{A_{f}E_{f}d}{s_{f}}$$

(9) $$K_{s}=\frac{A_{v}E_{s}d}{s}$$

where $E_{f}$ is the elasticity of the fabric. Assuming yielding of the shear reinforcement, the shear strength of the FRCM ($V_{f}$) is determined as follows:

(10) $$V_{f}=\frac{K_{f}}{K_{s}}V_{sy}$$

Finally, the shear strength is calculated using Equation (5), considering the following two cases:

(11) $$V_{s}=V_{sy}\;\mathrm{and}\;V_{f}=\kappa V_{fu}\;\mathrm{for}\;V_{f}<\kappa V_{fu}$$

(12) $$V_{s}=\frac{K_{s}}{K_{f}}V_{f}\;\mathrm{and}\;V_{f}=\kappa V_{fu}\;\mathrm{for}\;V_{f}\geq \kappa V_{fu}$$

**Figure 13:**
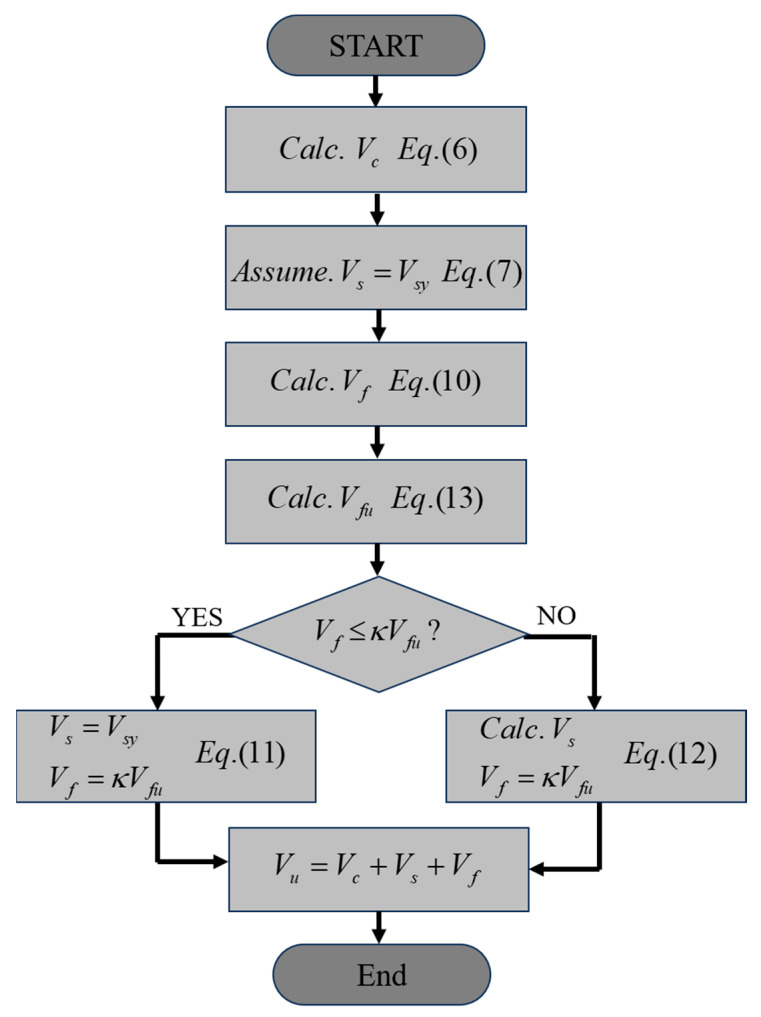
Flow chart.

The maximum shear strength of FRCM ($V_{fu}$) is calculated as follows:

(13) $$V_{fu}=\frac{A_{f}nf_{fu}d}{s_{f}}$$

where $A_{f}$ is the cross-sectional area of the fiber in the FRCM, n is the number of fabric layers, $s_{f}$ is the spacing of the fabric bundles, and $f_{fu}$ is the tensile strength of the fabric. In addition, κ is the bond reduction factor, which is introduced to reflect the partial bond capacity between FRCM and concrete substrates but does not reach the tensile strength ($f_{fu}$) of the fabric. κ is defined as a value ranging from 0 to 1. In $V_{f}<\kappa V_{fu}$, that is, Equation (11), the strengths of the concrete and shear reinforcement are maintained after the yielding of the shear reinforcement, and the shear strength of the beam is determined when the strength of the FRCM reaches its maximum value. However, in the case of the $V_{f}\geq \kappa V_{fu}$ in Equation (12), it is assumed that the maximum strength of the FRCM is developed before the yield strength of the shear reinforcement is reached; thus, the load-bearing capacity of the FRCM is instantaneously transferred to the shear reinforcement, and the FRCM-strengthened RC beam fails. Figure 13 illustrates the flowchart for determining the shear strength of a reinforced concrete beam with shear reinforcement strengthened by an FRCM. First, the shear strength of the concrete ($V_{c}$) was calculated, and the shear strength contribution of the FRCM ($V_{f}$) was calculated by assuming the yield of the shear reinforcement ($V_{s}=V_{sy}$). Then, depending on whether $V_{f}$ reaches $\kappa V_{fu}$, the shear strength of the shear reinforcement is determined using Equation (11) or Equation (12), and finally, the shear strength ($V_{n}$) is calculated by Equation (5).

### 4.2. Application of the Proposed Model 

**Figure 14:**
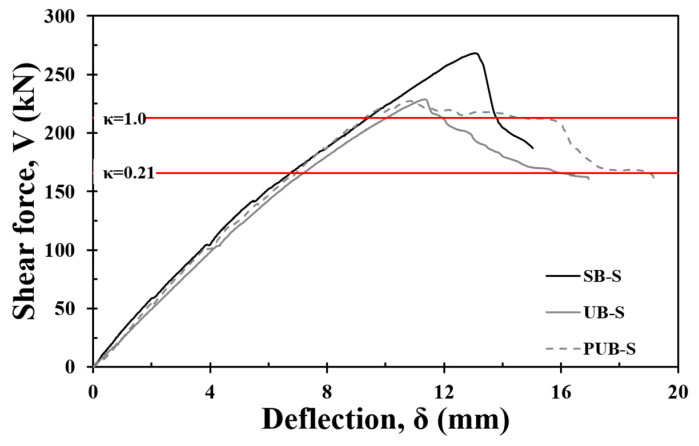
Evaluation of shear capacity considering ACI-318.

Figure 14 shows the results of the shear strength evaluation of the FRCM-reinforced specimens by applying the shear strength calculation model proposed in this study. When the value of the attachment reduction factor (κ) was adjusted from 0 to 1, the shear strength of the SB-S specimen was evaluated to be on the conservative side by 25% when κ = 1, and the shear strength of the UB-S specimen was similar to the shear strength calculated by applying κ = 1. If the bond reduction factor of κ = 0.21 is applied, the reinforcement does not yield when $V_{f}$ reaches $\kappa V_{f}$, as expressed in Equation (12), and the shear strength of the specimen was conservatively evaluated to be approximately 39%. This is similar to the calculated shear strength of the C-S specimen, which was determined by the sum of $V_{c}$ and $V_{sy}$ of Equation (6) and Equation (7), respectively; $V_{f}$ was not considered.

**Figure 15:**
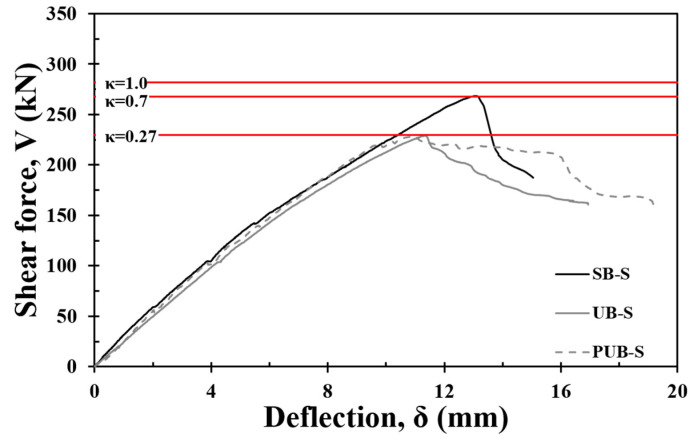
Evaluation of shear capacity considering test results.

As the shear strength of the test specimens was evaluated conservatively using the nominal strength provided by the design code, the shear strength of the concrete and shear reinforcement obtained from the test for the control specimens ($V_{c,test}$ and $V_{s,test}$) were applied to evaluate the effect of FRCM strengthening [37]. Subsequently, the bond reduction factor (κ), which accurately evaluates the experimental results, can be determined to indirectly evaluate the bond capacity between FRCM and concrete substrates. The shear strength of the C-NS specimen that was used for the $V_{c,test}$ and$V_{s,test}$ was determined as the difference between the shear strengths of the C-S and C-NS specimens obtained experimentally. As shown in Figure 15, a shear strength of 282 kN was calculated by applying κ = 1 under the assumption of a full bond. When the bond capacity was limited to 70% (κ = 0.7), the experimental shear strength of SB-S was 267 kN. When the bond capacity decreased to 27% (κ = 0.27), a shear strength of 229 kN was achieved in the UB-S specimen. As a result, it can be concluded that the SB-S specimen exhibits 70% of the maximum shear strength of the FRCM ($V_{fu}$), and the UB-S specimen exhibits 27% owing to the reduced bond capacity between the FRCM and concrete substrate. When applying the nominal strength of Equations (6) and (7) with κ = 0.7 and κ = 0.27 for SB-S and UB-S, respectively, their shear strengths were conservatively evaluated as 35% and 28%, respectively. 

## 5. Conclusions

In this study, RC beams strengthened by a fabric-reinforced cementitious matrix (FRCM) were experimentally investigated using the strengthening type as the main variable. DIC analysis was performed on the specimens, and the average shear strain, deflection contribution of the shear strain, and principal strain angle of the specimens subjected to shear force were compared. Subsequently, a shear strength evaluation model was proposed by introducing a bond reduction factor into the maximum shear strength of FRCM. The conclusions drawn from this study are summarized as follows.

(1) All the specimens failed with diagonal cracking within the shear span. The FRCM shear strengthening effect ranged from 14% to 65% in specimens without shear reinforcement. For the specimens with shear reinforcement, a shear strength improvement of 16% was confirmed in the FRCM-sided bond type; however, in the U-shaped strengthening specimen, it was difficult to confirm the strength improvement effect owing to the lack of bond capacity between the FRCM and concrete substrate, which caused the FRCM to fall off and the concrete to crush before the shear strength fully developed;
(2) For the PUB specimen strengthened using the prefabricated FRCM panel, the load was not transferred from the concrete member to the FRCM panel, and the strengthening effect could not be confirmed because the FRCM panel detached from the member. Therefore, the strengthening method should be improved to secure the bond capacity so that the FRCM and concrete substrate can exhibit fully bonded behavior;
(3) Based on the experimental data, the average shear strain data obtained through DIC displacement tracking were compared with the trend of the experimental data, and it was found that the shear stiffness tends to decrease after crack initiation, and FRCM strengthening can control the decrease in shear stiffness. In particular, U-shaped FRCM strengthening exhibited the best control of cracking and shear stiffness reduction;
(4) Based on the DIC displacement data, longitudinal, vertical, and shear strains were derived, and the principal strain angle was calculated using the strain data. The principal strain angles of the FRCM-strengthened specimens were lower. The results of the comparison of the crack pattern and the calculated principal strain angle showed a similar trend overall but were quantitatively quite different. In addition, the contributions of shear and flexure to the vertical deflection were analyzed, and it was confirmed that the deflection contribution due to shear increased after shear cracking occurred;
(5) A shear strength evaluation model for FRCM-strengthened RC beams was developed by applying the bond reduction factor, and the shear strengths of the specimens were evaluated to be 28–35% conservative. To apply the proposed model to the FRCM strengthening design, it is necessary to verify the strength evaluation model using more experimental data.

## Figures and Tables

**Table 1:** Material properties.

Concrete		Rebar			
$f_{ck}$ (MPa)	$f_{sp}$ (MPa)	Type	Modulus of Elasticity $E_{s}$ (GPa)	Yield Strength $f_{y}$ (MPa)	Ultimate Strength $f_{u}$ (MPa)
36.84	2.18	D10	200	483	621
		D13		526	635
		D19		557	701

**Table 2:** Properties of FRCM.

Mortar			Fabric					
Type	Compressive Strength $f_{cm}$ (MPa)	Tensile Strength $f_{tm}$ (MPa)	Type	Distance between Tows (mm)	Area $A_{f}$ (mm^2^)	Modulus of Elasticity $E_{f}$ (GPa)	Tensile Strength $f_{fu}$ (MPa)	$\varepsilon_{fu}$ (%)
C	-	-	Carbon	20	0.838	184	1962	1.07
SB	59.00	7.41						
UB	62.70	6.51						
PUB	41.58	5.86						

**Table 3:** Summary of test results.

Specimen	$V_{u}$ (kN)	$\delta_{u}$$\;\mathrm{at}\;V_{u}$ (mm)	$V_{cr}$ (kN)	$\delta_{cr}$$\;\mathrm{at}\;V_{cr}$ (mm)	$V_{u}$$-V_{u\_control}$ $(V_{f}$)
C-NS	93.3	4.04	93.3	4.04	0
SB-NS	135.3	5.78	134.4	5.62	42
UB-NS	153.8	8.14	112	4.96	60.5
PUB-NS	106.1	4.40	-	-	12.8
C-S	233.4	11.04	105	3.98	0
SB-S	268.0	13.08	141	5.56	34.6
UB-S	228.7	11.36	122.4	5.08	−4.7
PUB-S	227.5	10.82	-	-	−5.9

Note: $V_{u}$: ultimate shear strength, $\delta_{u}$: deflection at ultimate shear strength, $V_{cr}$: cracking shear strength, $\delta_{cr}$: deflection at cracking shear strength, $V_{f}$: contribution of FRCM.

## Data Availability

The original contributions presented in the study are included in the article, further inquiries can be directed to the corresponding author/s.
